# Patient-Specific Planning for Thermal Magnetic Resonance of Glioblastoma Multiforme

**DOI:** 10.3390/cancers13081867

**Published:** 2021-04-14

**Authors:** Eva Oberacker, Cecilia Diesch, Jacek Nadobny, Andre Kuehne, Peter Wust, Pirus Ghadjar, Thoralf Niendorf

**Affiliations:** 1 Berlin Ultrahigh Field Facility, Max-Delbrück-Center for Molecular Medicine in the Helmholtz Association, 13125 Berlin, Germany; cecilia.diesch@mdc-berlin.de (C.D.); thoralf.niendorf@mdc-berlin.de (T.N.); 2 Department Radiation Oncology, Charité–Universitätsmedizin Berlin, Corporate Member of Freie Universität Berlin and Humboldt-Universität zu Berlin, Augustenburger Platz 1, 13353 Berlin, Germany; jacek.nadobny@charite.de (J.N.); peter.wust@charite.de (P.W.); pirus.ghadjar@charite.de (P.G.); 3 Department of Physics, Faculty of Mathematics and Natural Sciences, Humboldt-Universität zu Berlin, 10117 Berlin, Germany; 4 MRI.TOOLS GmbH, 13125 Berlin, Germany; kuehne@mritools.de; 5 Experimental and Clinical Research Center, Joint Cooperation between Charité Unversitätsmedizin and the Max-Delbrück Center for Molecular Medicine in the Helmholtz Association, 13125 Berlin, Germany

**Keywords:** thermal magnetic resonance, radiofrequency hyperthermia, patient-specific therapy planning, EMF simulations, RF applicator, glioblastoma multiforme

## Abstract

**Simple Summary:**

Hyperthermia was proven to enhance the efficacy of chemo- and radiation therapy treatment of glioblastoma multiforme, an aggressive brain tumor of poor prognosis. Despite good clinical results in other tumor types and locations, hyperthermia induced by electromagnetic waves in the radiofrequency range is not available so far for the treatment of brain tumors due to the highly sensitive surrounding tissue and lack of non-invasive therapy monitoring. ThermalMR integrates non-invasive diagnosis, therapy, and therapy monitoring in a single RF applicator device by employing radiowaves for magnetic resonance imaging, radiofrequency heating, as well as magnetic resonance thermometry. This work examines three optimization algorithms for hyperthermia treatment planning and up to ten RF applicator configurations for a cohort of nine patient models with glioblastoma multiforme. Clinical diversity is represented in target size and location and the inclusion of post-operative models. Our findings indicate the need and potential for patient-specific treatment planning and RF applicator design when targeting brain tumors.

**Abstract:**

Thermal intervention is a potent sensitizer of cells to chemo- and radiotherapy in cancer treatment. Glioblastoma multiforme (GBM) is a potential clinical target, given the cancer’s aggressive nature and resistance to current treatment options. This drives research into optimization algorithms for treatment planning as well as radiofrequency (RF) applicator design for treatment delivery. In this work, nine clinically realistic GBM target volumes (TVs) for thermal intervention are compared using three optimization algorithms and up to ten RF applicator designs for thermal magnetic resonance. Hyperthermia treatment planning (HTP) was successfully performed for all cases, including very small, large, and even split target volumes. Minimum requirements formulated for the metrics assessing HTP outcome were met and exceeded for all patient specific cases. Results indicate a 16 channel two row arrangement to be most promising. HTP of TVs with a small extent in the cranial–caudal direction in conjunction with a large radial extent remains challenging despite the advanced optimization algorithms used. In general, deep seated targets are favorable. Overall, our findings indicate that a one-size-fits-all RF applicator might not be the ultimate approach in hyperthermia of brain tumors. It stands to reason that modular and reconfigurable RF applicator configurations might best suit the needs of targeting individual GBM geometry.

## 1. Introduction

Glioblastoma multiforme (GBM) is the most aggressive type of cancer and is still considered to be incurable. For GBM treatment, multimodal approaches including surgery and chemo- and radiation therapy in conjunction with adjunct hyperthermia therapy (HT) have proven to be of clinical benefit [1]. HT of various tumor types and locations using low (8–13.56 MHz), intermediate (70–100 MHz), or high (433 MHz) radio- frequencies (RFs) has culminated in a body of literature documenting the benefit of thermal intervention for boosting the efficacy of chemo- and radiation therapy [2,3,4,5,6,7,8]. Notwithstanding the encouraging results obtained for GBM treatment with interstitial hyperthermia [1], research into a less invasive approach is needed. These explorations include thermo-ablation via transcranial magnetic resonance (MR)-guided focused ultrasound, which demonstrated superb focal quality but limited ability to cover large target volumes [9,10,11]. Other approaches take advantage of modulated capacitive hyperthermia [12], which is constrained by insufficient specific absorption rates (SAR) and limited focusing in deep seated target regions [13,14]. Deep RF induced hyperthermia of GBM presents an alternative direction. Recent developments have reported on RF hyperthermia of GBM using fixed frequencies (f = 297 MHz [15,16], f = 915 MHz [17]) or multiple frequencies [18,19]. Magnetic nanoparticle heating has also shown promising results as localized adjuvant therapy [20,21]. All of these techniques can help to boost treatment efficacy by enhancing the permeability of the blood–brain–barrier for drugs or nanoparticles used in a combined treatment regime [22,23,24], with the most intriguing combination being the use of thermoresponsive carriers releasing the drugs only in the heating target site, allowing for a reduction of systemic side effects [25,26]. For the latter as well as for RF hyperthermia treatments in the brain, control over the RF power deposition in magnitude and spatial distribution is essential [27].

Taken together, this has stimulated the development of RF hardware including multi-channel signal generators and RF applicators tailored for HT of the brain [15,16,27,28,29]. Typical RF hyperthermia devices are annular-phased-arrays (APA) with the steer- ability of the RF field depending on the number of independent transmit channels [2,15,30]. Arranging the antennae in multiple rings in the cranial–caudal direction increases the focusing capabilities of the RF array [16,31,32]. Moreover, HT planning (HTP) procedures have been advanced using numerical approaches, including field shaping optimization algorithms [19,33,34]. A number of algorithms has been investigated, differing in constraints and targets used for optimization. Some algorithms target the global RF power deposition [33], optimize the dominant component of the electric (E-) field [34], use virtual observation points (VOPs) to accelerate the optimization [16], or compute an optimum fit to a given target RF power distribution using one or multiple RF frequencies [19]. These hardware and software developments are of relevance for brain tumor HT, where a rigorous confinement of high RF exposure levels to the target volume (TV) is essential. To enable efficient and safe RF power deposition, fulfillment of physical, engineering, and computational requirements is of the essence. Ensuring a patient and problem-oriented adaptation of the size, uniformity, and location of the RF energy de- position in the brain target region is highly relevant for HT, with the focal point quality being governed by the radiation pattern of the single RF transmit element, the RF channel count, antenna positioning and arrangement, and by the thermal intervention radio- frequency of the RF applicator. Assuming a brain matter ratio of approximately 40% white matter and 60% grey matter, f = 297 MHz results in a wavelength of ~14 cm and in a minimum achievable hotspot size of ~4.5 cm. This is in the range of realistic brain tumor TVs [35,36], which deems 297 MHz to be suitable for HT of GBM.

Dipole antenna building blocks are of proven value for HT applicators [15,16,18,28,31,37,38,39]. If assembled in APA, the long dipole axis is arranged in the cranial–caudal direction to maximize the component of the incident electric field, reaching the body surface tangentially to minimize superficial RF power deposition. This coincides with the dipole arrangement used for magnetic resonance imaging (MRI), where the magnetic component of the incident EM field is required to be perpendicular to the static magnetic field of the MR scanner, which is aligned in the cranial–caudal direction. Using an operational frequency of 297 MHz suits the physical requirements for RF focusing and matches the operational frequency of MRI at 7 Tesla. Thermal magnetic resonance (ThermalMR) is an HT variant that integrates RF-induced heating [16,27,39,40,41], in vivo temperature mapping using MR thermometry (MRT) for temperature dose and therapy control [27,42,43,44], anatomic and functional MRI for diagnosis and patient positioning, and the option for x-nuclei MRI in a single, multi-purpose RF applicator permitting supervised targeted temperature modulation [15,16,45].

GBM size and geometries range from small TVs challenging the focusing capabilities of an RF applicator as well as the field shaping algorithm to large TVs requiring a uniform distribution of RF energy in regions whose dimensions can reach a full wavelength. Notwithstanding the broad spectrum of patient specific GBM size and geometry, a sharp decline of RF exposure at the interface to surrounding healthy tissues is a key requirement for HTP. Recognizing the engineering challenges and the clinical context, this work examines the efficacy of RF field shaping for brain HT in nine clinically diverse GBM TVs. For this purpose, electromagnetic field (EMF) simulations that incorporate models based on clinical data obtained from computed tomography scans of GBM patients are performed. Three RF field shaping algorithms are applied to assess the performance of up to ten dipole building block-based RF applicator designs per patient model. For quantitative analysis, five metrics including (i) the maximum specific absorption rate averaged over 10 g tissue (SAR_10g_) in the TV, (ii) the SAR amplification factor (SAF), (iii) the relative target coverage where the RF power deposition exposure level exceeds the limit set for healthy tissue, (iv) the target-to-hotspot quotient (THQ) and (v) the volumetric power density in the TV are used. These (metrics) will be examined to identify the most successful HTP algorithm and RF applicator design. We hypothesize that the VOP-based power optimization outperforms the VOP-based uniformity optimization for RF field shaping [16], and that the iterative multiplexed vector field shaping (MVFS) approach provides a higher accuracy than the VOP-based approaches. We also expect the planar RF applicator configurations to yield modest HTP results while the high density RF applicators with a high channel count might not show improvement against RF applicators with lower channel count but the same head coverage. After testing our hypotheses we will draw conclusions on predictors of HTP outcome based on GBM target size and location.

## 2. Materials and Methods

### 2.1. Patient Voxel Models

**Figure 1:**
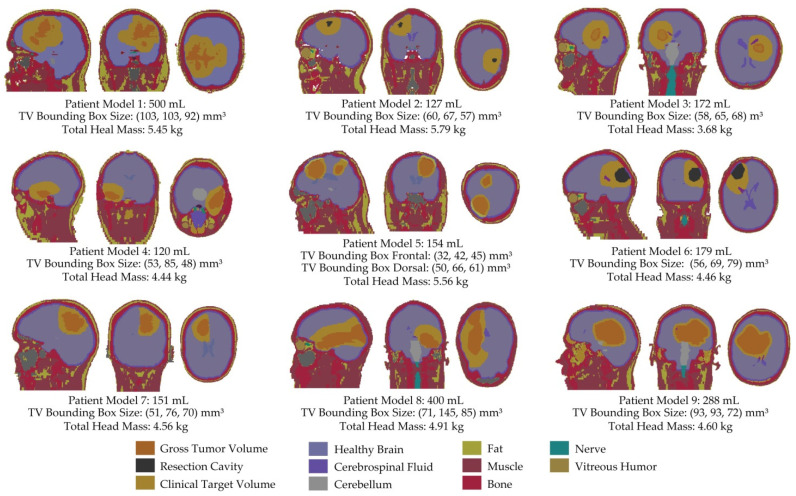
Nine head voxel models were generated based on clinical CT data derived from glioblastoma multiforme (GBM) patients. Each model is depicted in sagittal, coronal, and axial view with the slice positioned at the greatest extent of the target volume. The volume and the bounding box size are indicated for each patient model. The brain was homogeneously modeled (grey) and assigned the resulting dielectric properties (40% white matter + 60% grey matter; σ (avg brain) = 0.52 S/m, ε_r_ (avg brain) = 50.3). The macroscopic tumor mass (dark orange) was assigned σ (tumor) = 1.15 S/m, ε_r_ (tumor) = 66.5 [47]; the resection cavity (black) was assigned dielectric properties of blood (σ (blood) = 1.32 S/m, ε_r_ (blood) = 65.7); the clinical target volume surrounding the tumor (light orange) was assigned dielectric properties of the brain. The latter together with either the gross tumor volume or the resection cavity form the target volume for patient-specific hyperthermia treatment planning (HTP). In Pat Model 4, the tilting of the head results in the axial slice at the largest extent of the clinical target volume (CTV) to coincide with the CSF surrounding the frontal lobe of the brain. What looks like a seemingly disconnected spot of CSF behind the eyes is in fact a continuous layer to the slice above.

One aim of this study was to assess the feasibility of our HTP workflow [16] in a larger and more diverse set of clinical TVs. For this purpose, nine patient data sets (Pat Model 1–9) were selected for the creation of human voxel models used for EMF simulations [46]. Upon selection, we aimed for a high diversity in TV size and location, often favoring models deemed more challenging for successful treatment. The most challenging cases included very large TVs (Pat Model 1 and 8), small and superficial TVs (Pat Model 2 and 4), TVs with a small extent in the cranial–caudal direction (i.e., along the long axis of the dipole antenna; Pat Model 4), and even a model with two separate small TVs (Pat Model 5). To further broaden the representation of the clinical GBM spectrum, two patient models (Pat Model 2 and 6) were based on the postoperative CT scans of patients whose tumor was deemed operable. For these cases, the core of the TV did not consist of solid, macroscopic tumor tissue but a resection cavity filled with blood. Figure 1 illustrates all nine patient models using slices positioned at the greatest extent of the target volume together with their volume, bounding box size (i.e., maximum extent in all three dimensions) and the total mass of the simulated model.

Most organs and tissues were delineated for all patient models, while some specific organs at risk were selected based on the individual location of the target volume (see Appendix A). Three-dimensional voxel models with the resolution of the underlying CT scan were generated using a previously developed workflow [46]. Large ventricles filled with highly conductive cerebrospinal fluid (CSF, σ = 2.22 S/m [48,49]) were easily segmented from the organs at risk. Delineating the thin outer CSF layer surrounding the brain is more challenging, yet of importance to address the potential shielding effect by the closed layer. To address this challenge, we created this layer in the simulation model by recreating the shape of the brain with an isotropic size increase of 5%. The resulting layer was assigned dielectric properties of CSF and set to overwrite the adjacent fat/muscle/bone structure of the skull. For a full list of delineated organs, please refer to Appendix A.

### 2.2. Radiofrequency Applicators and Electromagnetic Field Simulations

The RF applicators used in this study were comprised of bow tie dipole antennae submerged in a high permittivity medium for wavelength and thus dipole length shortening. Heavy water (ε_r_ ≈ 81) [15] and a ceramic slurry (ε_r_ ≈ 200) [16] can be used to govern the size of the resulting building block and thus the number of independent channels being azimuthally arranged around the head. The RF applicator configurations differ in (i) number of independent transmit channels, (ii) coverage of the head in the cranial–caudal direction, (iii) azimuthal arrangement, and (iv) the presence/absence of a water bolus:

(i) The number of independent transmit channels was increased from 8 to 16 to 32. In the denomination of the RF applicators, this is represented at the first position: 8.*/16.*/32.*.
(ii) For the eight and 16 element arrays, the coverage in the cranial–caudal direction was increased in three steps and was encoded in the second position of the RF applicator denomination. Starting in a planar arrangement (*.P.*), first every second element was displaced by half the length of the dipole to obtain an interleaved arrangement (*.I.*), followed by shifting them by the full length of the RF antenna building block to form a two-row arrangement (*.2R.*).
(iii) For every RF applicator configuration of (i) and (ii), the influence of arranging the dipole building blocks in a ring (*.*.R; d = 240 mm) around the head versus an elliptical arrangement with the same circumference but better conformity to the human head (*.*.E; a_1_ = 220 mm, a_2_ = 260 mm) was investigated.
(iv) Adding a water bolus between the RF applicator and the patient’s body increases RF coupling and can serve to adjust/decrease the surface temperature [50]. The presence of a water bolus (*.WB) was tested for its influence on the treatment planning outcome for the interleaved arrays 16.I.*.WB.

**Figure 2:**
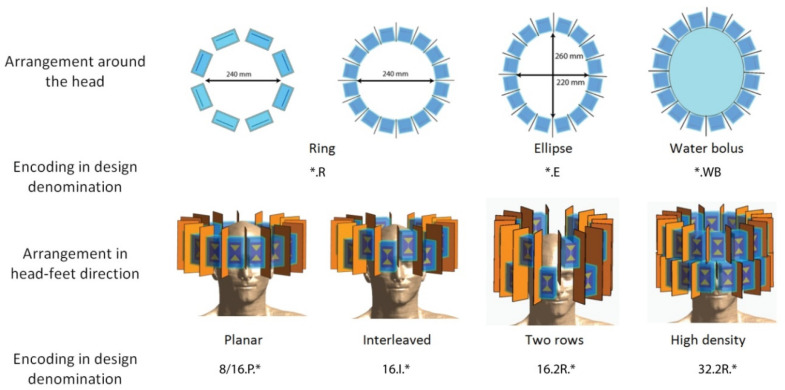
Schematic overview summarizing the RF applicator design elements (arrangement around the head, number of transmit elements and arrangement in the cranial–caudal direction, presence of a water bolus) and their encoding in the design denomination.

All RF applicator configurations are summarized in Figure 2.

The extensive EMF simulations of the 32 channel high density RF applicator designs were limited to the first three patient models, aiming at confirming our hypothesis that the improvement in HTP vs. the 16 channel RF applicators does not outweigh the doubled computational, engineering, and manufacturing effort [16]. Similarly, simulations of a water bolus were limited to the more promising ergonomic design 16.I.E.WB for five patient models.

For EMF simulations, the head voxel models were placed in the RF applicator with the center of the TV aligning with the center of the RF applicator in the cranial–caudal direction. In the other two dimensions, the head model was centered in the applicator.

All healthy organs were assigned dielectric properties, as listed in the IT’IS database [49]. Solid tumor tissue was assigned σ = 1.15 S/m and ε_r_ = 66.5, the average of two values reported from the only in vivo measurement known to the authors [47]. The clinical target volume (CTV) around the macroscopic tumor growth was assigned dielectric properties of healthy brain tissue (σ_avg brain_ = 0.52 S/m) due to unknown properties of microscopically infiltrated tissue. Additionally, no further information was available on the potential presence of edema in the CTV. In both cases, the assigned electric conductivity was lower than what could be suspected (σ_CSF_ = 2.22 S/m, σ_blood_ = 1.32 S/m, σ_tumor_ = 1.15 S/m). The calculated power deposition would thus potentially be underestimating the real SAR, making this a lower bound approach to the matter. With promising HTP results, this could indicate an even better treatment realization, should the real electric conductivity be higher than considered here. The same is true for the modeling of the resection cavity in Pat Models 2 and 6, were dielectric properties of blood are assumed, consisting of a lower bound approach to a potential mix of blood and CSF in the post-operative cavity. For a full list of delineated organs and assigned dielectric properties, please refer to Appendix A.

EMF simulations of the patient model + RF applicator configuration were performed for each individual transmit channel using Sim4Life V3.4 (ZurichMedTech, Zurich, Switzerland). Resolving the triangular shape of the dipoles (requires high resolution) yet maintaining a reasonable computation time (favors a low resolution) led to highly anisotropic gridding throughout the simulation volume. A minimal resolution of 3 × 3 × 3 mm^3^ was maintained within the brain. The results were exported for further processing in MATLAB (The MathWorks, Natick, MA, USA).

### 2.3. Hyperthermia Treatment Planning

From the channel-wise E-field data, the specific absorption rate was calculated, isotropically rebinned to a 3 × 3 × 3 mm^3^ resolution and finally averaged over 10 g of tissue (SAR_10g_) [51]. Our approach of ThermalMR includes HTP and MR imaging, and the 10 g averaging mass was thus chosen to address the requirements of RF hyperthermia and of MRI, where SAR_10g_ is used to monitor safe RF exposure limits in healthy tissue [52]. The SAR averaging was implemented using a previously described algorithm [53] to ensure that formulations violating the conservation of energy are not introduced. The power balance for all simulations was assessed as a standard QA precaution based on the formalism introduced in [54], and the worst-case power imbalance was within the bound- aries expected by the FDTD method. A good correlation of this averaging mass with temperature rise was reported for RF hyperthermia treatment of a human subject [55] in the head [56,57] and for the employed frequency range [51,58].

Based on SAR_10g_ data, patient-specific hyperthermia treatment planning was performed. For this purpose, our established optimization algorithms were employed to solve the problem in the semidefinite relaxation approximation of quadratic form maximization [16] using VOPs [59] to decrease computation time. Two approaches were used to either (i) directly maximize the total power delivered to the TV (VOP power optimiza- tion), or (ii) to address the homogeneity of the resulting RF power deposition in the TV as a second optimization goal (VOP uniformity optimization).

**Figure 3:**
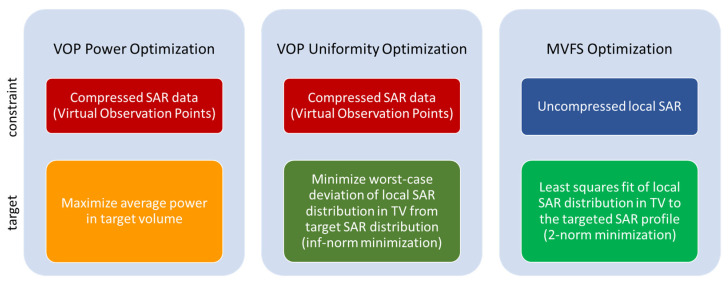
Common and different features of the VOP-based and multiplexed vector field shaping (MVFS)-based optimization algorithms based on the constraints and targets used to solve the optimization problem. The targeted SAR used in this study was a uniform exposure at 100 W/kg, pushing the optimization into a high SAR plateau, as analyzed in [60].

The use of VOPs decreased the computation time of the actual optimization. Computing the VOPs turned out to be the most time-consuming step in the post-processing. Additionally, VOP compression inherently requires a certain overestimation of the local SAR_10g_ data [59], which can only partly be removed by rescaling the field data after optimization. For these two reasons, the multiplexed vector field shaping (MVFS) approach was implemented in a second step, enabling a higher accuracy due to the use of the full SAR data and a shorter overall computation time enabled by its iterative approach [19]. Mathematically speaking, the VOP-based algorithms are special cases of the MVFS optimization problem using different constraints and targets to solve the problem, as summarized in Figure 3.

All optimization algorithms provably solve for the global optimum and inherently yield a set of excitation vectors that are to be played out in a time-interleaved manner, each indicating phase and amplitude for each individual transmit dipole antenna. The number of excitations is not chosen prior to the optimization, but is an additional outcome of the optimization process. The maximum number of excitations is equal to the number of individual elements in the RF array, but typically much lower for realistic application scenarios. The cumulative exposure of all vectors results in the most favorable overall SAR_10g_ exposure for the TV achievable with the optimization algorithm. A full mathematical treatment of the algorithm’s features and optimality can be found in the literature [19].

All optimization algorithms facilitate the definition of a local safe exposure level accepted in the healthy tissue. This level was set to 40 W/kg to limit the potential temperature increase to ΔT < +1.5 K [16] for all cases.

### 2.4. Performance Analysis

For the performance analysis of the RF applicators and of the HTP, five metrics were used for quantitative assessment. These metrics allow for the investigation of localized and averaged power deposition in the target volume as well as the healthy tissue. For all metrics, the general qualitative rule of thumb of “the higher the better” applies.

- The maximum SAR_10g_ value reached in the TV SAR_10g,max_(TV) assesses the absolute RF exposure level.
- The SAR amplification factor (SAF) [33] helps to quantify the sparing of the healthy surrounding tissue by referring the average power deposition in the TV to the average power absorption in the healthy tissue. However, SAF lacks information about the absolute SAR values reached. The SAF concept was introduced to specifically assess the feasibility of RF focusing on a small (ø 3 cm) target in the brain [33]. Furthermore, it lends itself to exploratory comparisons of different arrays because its global optimum can be straightforwardly computed. The best computationally possible SAF (ultimate SAF) achieved by optimal superposition of the E-fields emitted by a cluster of hundreds of dipoles arranged equidistantly around the head serves as a benchmark for HTP results. For deep seated targets, the best SAF reported at 298 MHz was 8.4. Please note that the SAF metric normalizes to the remaining healthy tissue as a whole without enforcing a limit on the local exposure. It is thus to be expected that this value cannot be reproduced for larger TVs, where the ratio between TV and healthy tissue is increased. It is calculated as SAF = SAR_10g,mean_(TV)/SAR_10g,mean_(healthy).
- The relative target coverage where the power deposition exposure level exceeds the limit set for the healthy tissue TC_SAR>Lim_ is a measure of the RF energy distribution within the TV. Since this in an investigative metric to judge our HTP outcome, no minimum requirement can be formulated yet. It is quantified as TC_SAR>Lim_ = V_TV_(SAR > SAR_lim_)/V_TV_.
- The target-to-hotspot quotient THQ [61] assesses local RF power deposition maxima in healthy tissue by correlating the average power deposition in the TV to the first volumetric percentile of local SAR_10g_ exposure in healthy tissue. We were the first to report a THQ > 1 in HTP of brain tumors [16,62]. THQ is calculated as THQ = SAR_10g,mean_(TV)/P_1,mean_(SAR_10g_(healthy)).
- The volumetric power density in the TV P_TV_/V_TV_ as a relative measure ensures the comparability of the results between different TVs and might help to differentiate TVs with a good treatment perspective from others. It relates the total power deposited in the TV P_TV_ to its volume V_TV_. This metric is particularly important, since the deposited power P_TV_ is driving the RF heating in the TV. Relating it to V_TV_ makes it comparable between patient models. For an approximated average tissue density of 1 g/cm³, this metric corresponds to the mean SAR in the TV. We hope that a P_TV_/V_TV_ > 30 W/L is the minimum we can achieve.

We did not include TC25% [61,63,64] commonly used for superficial tumors because our previous studies revealed a TC25% > 75% in all HTP results [16], which does not add value to the current evaluation.

## 3. Results

### 3.1. Optimization Algorithm Comparison

**Figure 4:**
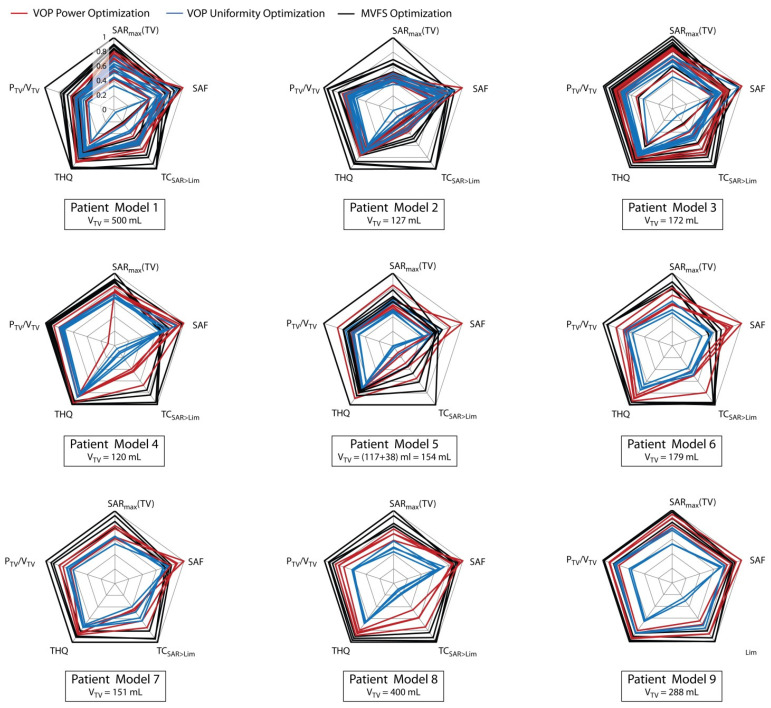
Comparison of all hyperthermia treatment planning results contrasted by optimization algorithm: red—VOP power optimization, blue—VOP uniformity optimization, black—MVFS optimization. All metrics are normalized to the respective inter-algorithm, inter-array maximum for each patient model.

First we compared the two VOP-based optimization algorithms for all patient models. Figure 4 depicts the optimization results for all possible configurations. All metrics were patient-wise normalized to the respective maximum across all algorithms (“inter-algorithm maximum”) and across all arrays (“inter-array maximum”) and plotted as radar plots, i.e., the better the optimization result, the further out the line will cross the axes. The optimal result would conform to the circumference of the plot.

Figure 4 suggests that the VOP power optimization (red) is always equal or superior to the VOP uniformity optimization (blue). In most cases, this is true for all metrics including the target coverage (TC_SAR>Lim_), indicating that trying to optimize the worst-case deviation from the target is an unsuitable optimization goal. VOP uniformity optimization was thus not further considered.

The plots also indicate that HTP using the MVFS algorithm (Figure 4, black lines) outperformed the VOP-based optimization algorithms. The only exception was Pat Model 9, where the VOP power optimization was competitive with the MVFS optimization. Interestingly, in all patient models, the SAF of the MVFS optimization underperformed compared to the VOP power optimization results. This is not as surprising, as only local SAR but not total absorbed RF power inside healthy tissues (i.e., global SAR) was constrained in any of the approaches. The MVFS algorithm managed to supply additional power to the target region at the expense of a higher average power density in healthy tissues, as it was more efficient in exploiting power deposition in healthy tissue just below the local SAR threshold of 40 W/kg. Given that all optimization algorithms use the same constrained safe local RF exposure limit in healthy tissue, this is considered negligible considering the significant improvement obtained for all other metrics. If desired, total power deposition inside healthy tissues could be included in the constraints as well. From here on, we thus focused on the MVFS algorithm.

Table 1 summarizes the largest value achieved for each metric and patient model and outlines which RF applicator design and optimization algorithm was used. For each of the listed optimization result, the number of excitations resulting from the HTP is indicated. These phase and amplitude settings are to be executed in a time-interleaved manner for the same duration (i.e., two excitation vectors = half the treatment time for each phase and amplitude excitation setting). By fast switching between the excitation settings, the cumulative exposure then results in their averaged SAR pattern, as obtained by the HTP. While the most favorable RF applicator design changed throughout the patient models as well as the metrics in comparison, the MVFS optimization outperformed the other optimization algorithms for all patients and metrics except the SAF, where the VOP power optimization yielded the best results. For most of the best results found with the MVFS optimization, a combination of two excitations was found sufficient for highly targeted RF exposure. Only for Pat Models 5 and 9 was a set of three excitation patterns more successful. Especially for the first, this is easily understandable given the challenging geometry of the split TV. The best SAF results obtained using the VOP power optimization required just one excitation. For the full results, please refer to the Supplementary Materials.

For each metric, we calculated the mean, standard deviation, and median of the best results possible with our RF applicators and optimization algorithms to relate them to our HTP aims: SAR_10g,max_ = (110.3 ± 22.1) W/kg, median = 114.4 W/kg; SAF = 4.0 ± 1.0, median = 3.8; TC_SAR>Lim_ = (78.1 ± 14.2)%, median = 83.1%; THQ = 1.4 ± 0.2, median = 1.4 > 1; P_TV_/V_TV_ = (49.4 ± 8.7) W/L, median = 49.1 W/L >> 30 W/L. Since these data contain clinically unrealistic combinations of multiple RF applicators and/or optimization algorithms for the same patient model, we repeated the same analysis for one configuration, choosing the RF applicator and optimization algorithm most frequently yielding the best HTP result for each patient model individually: SAR_10g,max_ = (110.3 ± 22.1) W/kg, median = 114.4 W/kg; SAF = 3.3 ± 0.6, median = 3.3; TC_SAR>Lim_ = (77.1 ± 14.0)%, median = 76.5%; THQ = 1.4 ± 0.2, median = 1.4 > 1; P_TV_/V_TV_ = (49.4 ± 8.7) W/L, median = 49.1 W/L >> 30 W/L.

### 3.2. RF Applicator Comparison: Number of Transmit Channels

**Figure 5:**
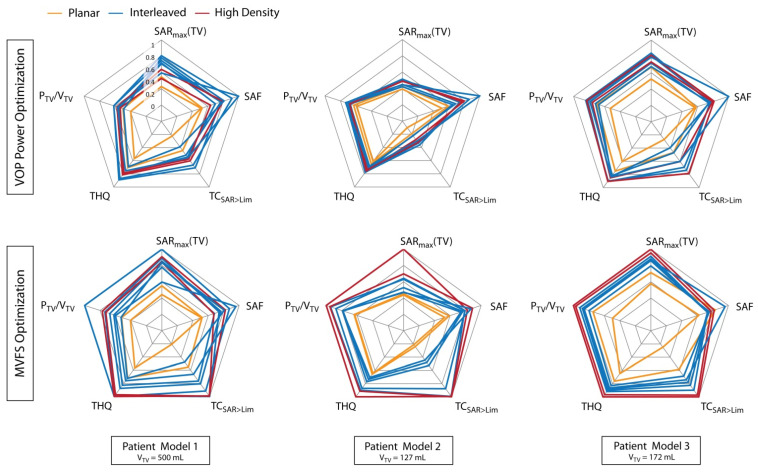
Influence of the number of independent transmit channels and their arrangement on the TIP results, comparing the VOP power optimization and the MVFS optimization. For better comparability, all metrics have been normalized to their inter-algorithm, inter-array maximum. Orange: planar arrays, *.P.*; blue: interleaved arrays, 16.I.* and 16.2R.*; red: high density arrays 32.2R.*.

With this part of the multi-model study, we aimed to draw a conclusion on the best suited RF applicator configurations with regard to the number of individual transmit elements and the head coverage along the cranial–caudal direction. Our preliminary findings (Pat Model 1) indicated that the planar designs (*.P.*) of the applicator provided insufficient degrees of freedom for field shaping, while the high density RF arrays (32.2R.*) underperformed. Extending our quantitative analysis to the Pat Models 2 and 3, these preliminary findings were confirmed when using the previously employed VOP power optimization (top row in Figure 5). However, for the MVFS optimization (bottom row in Figure 5), the outcome was not as conclusive. While the planar arrangements (orange lines) still clearly underperformed, the high density RF arrays (red lines) underperformed in Pat Model 1 but outperform all others clearly in Pat Model 2 and slightly in Pat Model 3, allowing for no definitive conclusion at this point. Comparing the 32.2R.* HTP results with the best results obtained with a 16.* RF applicator design for the most relevant metric P_TV_/V_TV_, the drop when moving from 32.2R.* to 16.2R.* was only −5.5% and −8.3% for Pat Models 2 and 3, respectively. In both cases, P_TV_/V_TV_ stayed above our goal of 30 W/L (P_TV_/V_TV_ = 37.8 W/L for Pat Model 2 and P_TV_/V_TV_ = 48.7 W/L for Pat Model 3). Given the great amount of engineering, manufacturing, and operating effort of the 32 channel RF arrays and power supply together with the modest results obtained for Pat Models 1 and 3 using the 32.2R.* designs, our further analysis focused on the 16 channels interleaved and two row designs (16.I.*/16.2R.*).

### 3.3. Hyperthermia Treatment Planning

**Figure 6:**
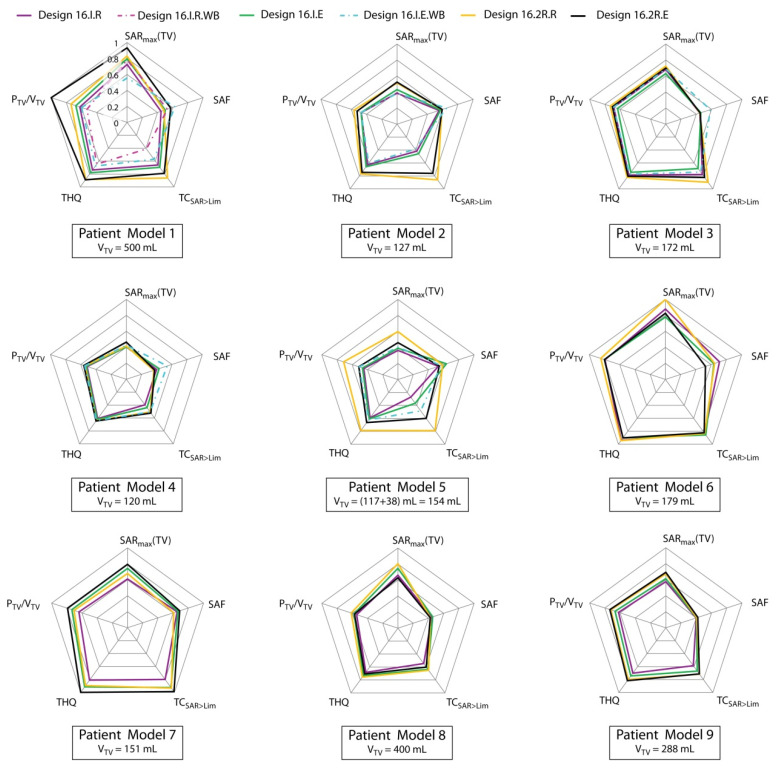
Comparison of the 16 channel interleaved and two row arrays (16.I.* and 16.2R.*) for all patient models. For better comparability, all metrics were normalized to their overall maximum. Purple: 16.I.R; magenta (dashed): 16.I.R.WB; green: 16.I.E; cyan (dashed): 16.I.E.WB; yellow: 16.2R.R; black: 16.2R.E.

For all nine patient models, the four 16 channel designs 16.I.* and 16.2R.* were simulated and evaluated. For Pat Models 1–5, a water bolus was incorporated in design 16.I.E.WB. The circular counterpart (16.I.R.WB) was simulated only for Pat Model 1. The goal of the thorough comparison of the HTP results was not only to learn about the suitability of the RF applicator designs but also to correlate the planning outcome with target size and location. For this purpose, the metrics displayed in Figure 6 were normalized to the overall inter-array maximum, making the plots comparable across all patient models. Please note that normalization to the overall maximum means normalizing the SAF to the value achieved using the VOP power optimization. In this figure displaying the results obtained using the MVFS optimization, no result reached maximum SAF.

We repeated our analysis for the mean, standard deviation, and median for all metrics when only taking the 16.* RF applicator designs into account: SAR_10g,max_ = (104.4 ± 24.4) W/kg, median = 104.3 W/kg; SAF = 3.1 ± 0.6, median = 3.3; TC_SAR>Lim_ = (75.9 ± 12.7)%, median = 76.5%; THQ = 1.4 ± 0.2, median = 1.3 > 1; P_TV_/V_TV_ = (48.7 ± 8.9) W/L, median = 48.7 W/L >> 30 W/L.

**Figure 7:**
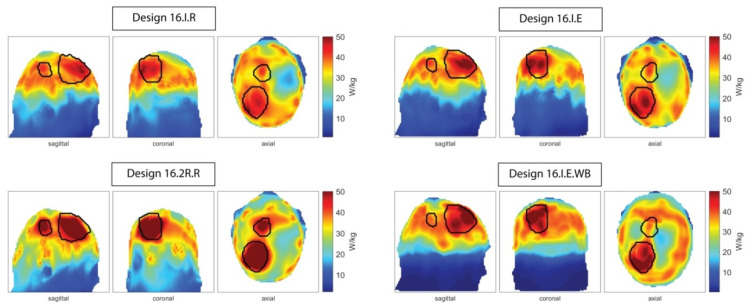
Maximum intensity projections of the SAR_10g_ distribution in Patient Model 5 obtained using the MVFS optimization with designs 16.I.R, 16.I.E, 16.2R.R, and 16.I.E.WB. These RF exposure patterns were achieved by the cumulative exposure of 3/3/3/2 excitation settings to be played out in a time-interleaved manner. Design 16.2R.R supports successful treatment planning of both sub-TVs with a single optimization run, enabling HT of both sub-TVs in a single session rather than two separate sessions requiring full cooling of the brain for accurate treatment prediction.

The HTP results for Pat Model 1 improved steadily through the iterations of the applicator design development; solely adding the water bolus did not improve the planning outcome. With P_TV_/V_TV_ = 66.8 W/L, the overall highest power density could be achieved in this very large TV using Design 16.2R.E for this patient model. For Pat Model 2, a significant improvement was found when moving from the interleaved designs 16.I.* to the two row arrangements 16.2R.*. This tendency persists throughout all patient models with either Design 16.2R.R or 16.2R.E showing the overall best performance. This is particularly interesting for the split target volume in Pat Model 5, where Design 16.2R.R was the only design supporting RF focusing on both targets simultaneously, allowing treatment of both tumor locations in a single session. The resulting SAR_10g_ distributions obtained for designs 16.I.R, 16.I.E, 16.I.E.WB, and 16.2R.R were depicted as maximum intensity projections in Figure 7. The overall highest SAR_10g,max_(TV) = 143.9 W/kg was obtained in Pat Model 6, a large superficial post-operative target. This could in part be explained by the slightly higher conductivity of blood vs. tumor. For the same model, the overall highest SAF of 5.8 was achieved by using the VOP power optimization and Design 16.2R.R. The highest SAF obtained using the MVFS optimization was 4.12 using Design 16.I.R for the same patient model. The HTP for Pat Model 7 attained both the highest THQ = 1.64 and the best target coverage among these designs with TC_SAR>Lim_ = 95.8%. The overall best coverage of 96.9% was achieved using Design 32.2R.R for Pat Model 3.

## 4. Discussion

This study carefully examined the applicability of RF applicator configurations and optimization algorithms for patient specific planning of targeted RF power deposition in the brain for a diverse set of realistic target volumes derived from GBM patient data. Our EMF simulations revealed the insufficient performance of the VOP uniformity optimization. MVFS optimization outperformed the VOP power optimization with the exception of SAF. This indicates that the better HTP results obtained with MVFS optimization results in higher average RF exposure in healthy tissue, by enabling a better RF distribution in the healthy tissue and thus a higher RF exposure of the TV. Since all optimization algorithms are constrained to the same local safe exposure limits in the healthy tissue, this is of minor impact considering the significant improvement of all other treatment metrics. The use of the MVFS optimization is thus recommended over VOP uniformity optimization and VOP power optimization, as hypothesized at the beginning of this study. Total power deposition inside healthy tissues could be included in the constraints as well. Our results were all obtained using a flat target exposure profile of 100 W/kg in the TV. Since the algorithm actually supports weighing of different regions by using inhomogeneous profiles [19], our work does not leverage the full potential of the algorithm, leaving room for further improvement by patient-specific TV definition, including areas subject to a boosted exposure, such as macroscopic tumor growth vs. margin used in the definition of the clinical target volume.

Comparison of our optimization algorithms to existing optimization algorithms from the literature was performed vs. an SAF optimization algorithm developed for TVs in the brain [33] and the FOCO algorithm developed for TVs in the head and neck region [34]. For our intents and purposes, the MVFS algorithm clearly outperformed the SAF optimization in all metrics but the SAF and performed at least as well if not better than the FOCO algorithm [19,65].

All results demonstrate a clear inferiority of the planar array designs 8.P.R and 16.P.R, as expected. Including the MVFS optimization algorithm into the evaluation of the number of independent transmit elements best suited for HT of GBM showed that the high density arrays perform better than assumed in the design of this study [16] and are superior to the 16 channel counterparts. These results were questioned by the data derived from VOP power optimization comparing all designs for Pat Models 1–3, which showed no superiority of the high density arrays 32.2R.*. Weighing the significant increase in engineering and manufacturing efforts required to set-up the high density 32 channel RF applicator against the insignificant changes in HTP outcome would lower the priority of an RF applicator design equipped with 32 transmit elements. To make ultimate conclusions and to define the priority on the number of independent transmit elements, further research is warranted involving a larger cohort of GBM patients. Regardless of the conclusion drawn on the RF applicator design, this finding also emphasizes the importance of a good HTP algorithm, leaving the potential of a better treatment outcome untapped otherwise.

Considering head coverage along the head–feet direction, the two row configurations 16.2R.* outperformed the versions with less cranial–caudal coverage, including the interleaved configuration 16.I.*. The enhanced head coverage of the 16.2R.* configurations was expected to benefit TVs with a large extent in the cranial–caudal direction (Pat Models 1, 6, and 8). While the expectations were met for Pat Models 1 and 6, no HTP improvement was obtained for Pat Model 8. This was contrasted by HTP improvements in the small Pat Models 2 and 5. Pat Model 4, however, showed again no HTP improvement despite almost equaling Pat Model 2 in volume. The commonality between Pat Model 4 and Pat Model 8, both with comparatively poor HTP outcomes, is the imbalance between the axial and radial extent of the TV. For the small TV of Pat Model 4, this can be easily understood by analyzing the RF power deposition pattern of an APA-applicator using λ/2 dipole antennas, where the field maximum extends along the long axis of the antenna. Even the two row arrangements of RF applicator models *.2R.* do not support sufficient focusing in the cranial–caudal direction to confine the SAR maximum to the small extent of the TV. For Pat Model 8 with a comparatively large extent in the cranial–caudal direction (≈2/3 λ), we expected better results given the nature of our HTP. With an optimization algorithm tailored for time-multiplexed RF exposure using multiple phase and amplitude settings at a rather small wavelength, our approach affords a successful cumulative RF exposure by sweeping a large TV with multiple separate maxima. Our results, however, suggest that TVs with a large imbalance between the axial and the radial extent remain challenging for HTP. This motivates further research into the feature of using multiple RF frequencies HTP since the MVFS algorithm adapts the main frequency based on the TV size [19]. For single frequency HTP, however, this comparison of TV dimensions could a priori indicate successful HTP and thus serve as an inclusion criterion for patient eligibility.

In addition, the TV location rather than the TV size might add to a better performance of the two row arrays for small TVs. The TV in Pat Models 2 and 5 are located directly underneath the skullcap. Since the dipole antenna building blocks are equally distributed in the axial plane to ensure transmission uniformity in MRI, no dipole elements are placed directly above the TV. Radiating the RF energy from a steeper angle rather than parallel to the skullcap seems to have a positive impact on the enhanced performance of the two row 16.2R.* RF applicator configuration. To test this hypothesis, simulations with the interleaved 16.I.* designs shifted in the caudal direction could provide further insights. However, design 16.2R.R afforded successful HTP of a split GBM TV (Pat Mod 5) and enabled treatment in a single session rather than two separate applications separated by a cooling period—which supports the applicability and efficacy of this design. Additionally, deep seated TVs (for example Pat Models 3 and 9) are favored because all RF elements can contribute almost equally to the RF exposure in the TV.

Using a circular versus an elliptical arrangement of dipole building blocks might have an impact on HT efficacy. The elliptical design proved superior to the circular arrangement for Pat Models 1, 2, 4, 7, and 9. This finding did not hold true for Pat Models 3, 5, 6, and 8. This indicates that a one-size-fits-all RF applicator might not be the ultimate solution for patient-specific HTP of brain tumors. This approach is commonly and successfully used in many RF applicators targeting other regions of the body using the APA technique [30,32,38]. These applications outside of the brain however always rely on patient feedback during treatment to detect and respond to local hot spots and patient discomfort [66,67,68]. Due to the lack of pain sensation in the brain [69], the same feedback approach is not feasible for HT of brain tumors. HT planning and prediction should thus be as precise as possible with as little trade-off for engineering or other reasons as possible, potentially requiring a decision between a circular or elliptical design on a patient-to-patient basis. This is in alignment with reports on choosing the position of the RF antennas within the RF array on a patient-to-patient basis for microwave HT of brain tumors [70].

This study demonstrated the value and power of numerical simulations and optimization algorithms for HTP in the brain. It is a recognized limitation of the study that (patho)physiological heat transport mechanisms including blood perfusion or thermo- regulatory mechanisms were not included in the simulations. Relying on the RF power deposition in tissue as measured by the SAR is very well established due to the high level of potential inaccuracies in thermal simulations in the body. However, using SAR instead of temperature might be viewed as a limitation of our study.

En route to careful validation of our simulations with experiments, we have developed an anthropomorphic head phantom, which mimics healthy brain tissue and brain tumor tissue [71]. To implement the experimental setup required for the validation, our group is currently advancing the installation of a high RF power transmit chain at our 7 T MRI system. We have already successfully implemented a transmit/receive switch supporting high average power for RF heating as well as very low power signals for MR imaging [41], a 32 channel RF signal generator tailored to ThermalMR [27], and an RF supervision module for phase and power monitoring [72]. We are very much looking forward to performing RF heating experiments with a high channel count.

## 5. Conclusions

This work elucidated the efficacy of planar and three-dimensional RF applicator configurations in conjunction with RF field shaping optimization for patient specific HTP in GBM target volumes. Our results demonstrate that for each patient model, HTP could be successfully performed, and an optimization algorithm for further use was identified. Minimum requirements formulated for the metrics investigated were met and exceeded for all patient specific cases.

To summarize, our findings add to the literature by underlining that a one-size-fits-all RF applicator is not the ultimate approach for HT of brain tumors. While a two row RF applicator arrangement provided best performance, it stands to reason that modular and reconfigurable RF applicator configurations might best suit the needs of targeting individual GBM geometry. To conclude, this research provides important data for patient specific HTP, with the emphasis on a sophisticated HTP algorithm, and forms a technological basis for future development of RF applicators tailored for ThermalMR applications in the brain. Our simulations are a mandatory precursor of broader experiments that are tailored to evaluate and validate RF applicator configurations en route to clinical application.

## Figures and Tables

**Table 1:** Summary of the highest values achieved for each patient model and metric together with the RF applicator design, optimization algorithm used, and number of excitations to yield the calculated RF exposure patterns by time-interleaved application. The overall maxima are highlighted in green italic. For the full table of HTP results, please refer to the Supplementary Materials.

Patient Model	Metric	Highest Value	Design	Algorithm	Number of Excitations
Pat Model 1 (V = 500 mL)	SAR_10g,max_ (W/kg)	134.2	16.2R.E	MVFS optimization	2
	SAF	3.8	16.I.E.WB	VOP power optimization	1
	TC_SAR>Lim_ (%)	83.5	16.2R.R	MVFS optimization	2
	THQ	1.5	16.2R.E = 32.2R.E	MVFS optimization	2/3
	P_TV_/V_TV_ (W/L)	*66.8*	16.2R.E	MVFS optimization	2
Pat Model 2 (V = 127 mL)	SAR_10g,max_ (W/kg)	117.5	32.2R.E	MVFS optimization	2
	SAF	4.3	16.I.E.WB	VOP power optimization	1
	TC_SAR>Lim_ (%)	84	32.2R.R	MVFS optimization	2
	THQ	1.4	32.2R.E	MVFS optimization	2
	P_TV_/V_TV_ (W/L)	40	32.2R.E	MVFS optimization	2
Pat Model 3 (V = 172 mL)	SAR_10g,max_ (W/kg)	114.4	32.2R.R	MVFS optimization	2
	SAF	3.5	16.I.E.WB	VOP power optimization	1
	TC_SAR>Lim_ (%)	*96.9*	32.2R.R	MVFS optimization	2
	THQ	1.5	32.2R.R	MVFS optimization	2
	P_TV_/V_TV_ (W/L)	53.1	32.2R.R	MVFS optimization	2
Pat Model 4 (V = 120 mL)	SAR_10g,max_ (W/kg)	66.9	16.2R.E	MVFS optimization	2
	SAF	3.2	16.I.E.WB	VOP power optimization	1
	TC_SAR>Lim_ (%)	50.8	16.2R.E	MVFS optimization	2
	THQ	1.1	16.2R.E	MVFS optimization	2
	P_TV_/V_TV_ (W/L)	37.4	16.2R.E	MVFS optimization	2
Pat Model 5 (V = 117 + 38 mL)	SAR_10g,max_ (W/kg)	86.1	16.2R.R	MVFS optimization	3
	SAF	5.2	16.I.E.WB	VOP power optimization	1
	TC_SAR>Lim_ (%)	76.5	16.2R.R	MVFS optimization	3
	THQ	1.3	16.2R.R	MVFS optimization	3
	P_TV_/V_TV_ (W/L)	47.7	16.2R.R	MVFS optimization	3
Pat Model 6 (V = 179 mL)	SAR_10g,max_ (W/kg)	*143.9*	16.2R.R	MVFS optimization	2
	SAF	*5.8*	16.2R.R	VOP power optimization	1
	TC_SAR>Lim_ (%)	83.1	16.I.E	MVFS optimization	2
	THQ	1.6	16.2R.R	MVFS optimization	2
	P_TV_/V_TV_ (W/L)	56.8	16.2R.R	MVFS optimization	2
Pat Model 7 (V = 151 mL)	SAR_10g,max_ (W/kg)	114.3	16.2R.E	MVFS optimization	2
	SAF	4.9	16.2R.E	VOP power optimization	1
	TC_SAR>Lim_ (%)	95.8	16.2R.E	MVFS optimization	2
	THQ	*1.6*	16.2R.E	MVFS optimization	2
	P_TV_/V_TV_ (W/L)	52.8	16.2R.E	MVFS optimization	2
Pat Model 8 (V = 400 mL)	SAR_10g,max_ (W/kg)	115.8	16.2R.R	MVFS optimization	2
	SAF	2.9	16.I.R	VOP power optimization	2
	TC_SAR>Lim_ (%)	63.1	16.2R.R	MVFS optimization	2
	THQ	1.2	16.2R.R	MVFS optimization	2
	P_TV_/V_TV_ (W/L)	41	16.2R.R	MVFS optimization	2
Pat Model 9 (V = 288 mL)	SAR_10g,max_ (W/kg)	99.7	16.2R.E	MVFS optimization	3
	SAF	2.7	16.2R.E	VOP power optimization	3
	TC_SAR>Lim_ (%)	69.3	16.2R.R	MVFS optimization	3
	THQ	1.3	16.2R.E	MVFS optimization	3
	P_TV_/V_TV_ (W/L)	49.1	16.2R.E	MVFS optimization	3

## Data Availability

Not applicable.

## Supplementary Materials

The following are available online at https://www.mdpi.com/article/10.3390/cancers13081867/s1, Table S1: Full VOP power optimization HTP results, Table S2: Full VOP uniformity optimization HTP results, Table S3: Full MVFS optimization HTP results.

## Appendix A

**Table A1:** Segmented target volumes and organs at risk with their assigned tissue types and dielectric properties for EMF simulations. Dielectric properties of healthy tissue were taken from the IT’IS database [48,49]. Solid tumor tissue was assigned the average of two values reported from the only in vivo measurement known to the authors [47]. Some organs and tissues were delineated for all patient models, while specific organs at risk were selected based on the individual location of the target volume. * Upon further discussion throughout the patient model creation, changes in the assigned dielectric properties were made, for example addressing the fact that cavities in the human head are typically not empty and should thus not be simulated as pure air. Comparison studies were performed (data not shown here) and showed no significant influence, even for the fairly large difference in conductivity and relative permittivity in the cavities. This is most likely due to the small geometrical extent and the comparatively large SAR averaging volume. ** The manual organ contouring sometimes generated residual voxels not assigned to any specific organ. These voxels were assigned averaged properties of muscle and fat.

Patient Model	Organ	Assigned Tissue	Electric Conductivity σ (S/m)	Relative Permittivity ε_r_
**All**	Brain	40% WM + 60% GM	0.52	50.3
	Brainstem	40% WM + 60% GM	0.52	50.3
	Eye	Vitreous humor	1.52	69
	Optical nerve	Nerve	0.42	37
	Chiasm *	White matter	0.42	43.8
	Spinal cord	Nerve	0.42	37
	Ventricles	CSF	2.22	72.8
	External CSF	CSF	2.22	72.8
	Fat	Fat	0.07	11.7
	Muscle	Muscle	0.77	58.2
	Skull	Bone	0.08	13.5
	Cavities *	Lung (inflated)	0.36	24.8
**Pat Model 1**	Tumor growth	Tumor	1.15	66.5
	Vessels	Blood	1.32	65.7
	Pituitary gland	Hypothalamus	0.69	60.1
	Hypothalamus	Hypothalamus	0.69	60.1
	Chiasm *	Nerve	0.42	37
	Cavities *	Air	0	1
**Pat Model 2**	Resection cavity	Blood	1.32	65.7
	Body **	50% Muscle + 50% Fat	0.42	35
	Hypothalamus	Hypothalamus	0.69	60.1
	Blood vessels	Blood	1.32	65.7
**Pat Model 3**	Tumor growth (solid)	Tumor	1.15	66.5
	Tumor growth (non-solid)	Tumor	1.15	66.5
	Body	50% Muscle + 50% Fat	0.54	42.7
	Hypothalamus	Hypothalamus	0.69	60.1
	Blood vessels	Blood	1.32	65.7
**Pat Model 4**	Tumor growth	Tumor	1.15	66.5
	Trepanation	Skin	0.64	49.9
	Hypophysis	Hypophysis	0.85	62.5
	Inner ear	60% Cartilage + 30% bone + 5% cavity + 5% muscle	0.45	39.2
**Pat Model 5**	Tumor growth	Tumor	1.15	66.5
	Inner ear	60% Cartilage + 30% bone + 5% cavity + 5% muscle	0.45	39.2
	Hippocampus	Hippocampus	0.69	60.1
	Lacrimalis	Thyroid Gland	0.85	62.9
	Cerebellum	40% WM + 60% GM	0.52	50.3
**Pat Model 6**	Resection cavity	Blood	1.32	65.7
	Inner ear	60% Cartilage + 30% bone + 5% cavity + 5% muscle	0.45	39.2
**Pat Model 7**	Tumor growth	Tumor	1.15	66.5
	Inner ear	60% Cartilage + 30% bone + 5% cavity + 5% muscle	0.45	39.2
	Hypophysis	Hypophysis	0.85	62.5
**Pat Model 8**	Tumor growth	Tumor	1.15	66.5
	Pituitary gland	Hypophysis	0.85	62.5
	Inner ear	60% Cartilage + 30% bone + 5% cavity + 5% muscle	0.45	39.2
**Pat Model 9**	Tumor growth	Tumor	1.15	66.5
	Inner ear	60% Cartilage + 30% bone + 5% cavity + 5% muscle	0.45	39.2
