# Supplementary material for: Patient-Specific Planning for Thermal Magnetic Resonance of Glioblastoma Multiforme
Source: Cancers (Basel). 2021 Apr 14;13(8):1867. doi: 10.3390/cancers13081867 (PMC8070230; doi:10.3390/cancers13081867)
Supplement: Supplementary file 1 [file cancers-13-01867-s001.pdf]

cancers

MDPI

Article

Supplementary Materials: Patient-Specific Planning for Thermal Magnetic Resonance of Glioblastoma Multiforme

Eva Oberacker, Cecilia Diesch, Jacek Nadobny, Andre Kuehne, Peter Wust, Pirus Ghadjar and Thoralf Niendorf

Supplementary Tables. Full hyperthermia treatment planning results for all patient models, RF applicator designs and optimization algorithms. The highest value of each metric per patient model and optimization algorithm is highlighted in bold. The inter-algorithm maximum is further highlighted by green font. The overall (inter-algorithm and inter-patient) maximum is further highlighted by a green background.

Table S1. Full VOP power optimization HTP results.

		VOP Power Optimization									
		**8.P.R**	**16.P.R**	**16.I.R**	**16.I.E**	**16.2R.R**	**16.2R.E**	**32.2R.R**	**32.2R.E**	**16.I.R.WB**	**16.I.E.WB**
**Pat Model 1**	$SAR_{max}(TV)$	57.3	73.4	96.4	94.8	100.3	**108.3**	70.7	85.6	104.6	79.9
	SAF	2.0	2.0	2.7	2.9	2.9	3.5	2.4	3.0	3.1	3.8
	$TC_{SAR>Lim}$	18.8	37.2	45.5	43.0	**59.1**	55.6	47.9	50.2	32.5	46.3
	THQ	0.8	1.0	1.2	1.2	**1.3**	1.3	1.2	1.2	1.0	1.1
	P_{TV}/V_{TV}	26.5	33.2	36.7	36.1	**41.2**	**41.2**	35.6	38.4	32.6	37.8
	# of excitations	2	2	2	1	1	2	2	2	1	1
**Pat Model 2**	$SAR_{max}(TV)$	50.0	46.1	49.6	52.7	58.7	**60.5**	57.6	57.4		52.3
	SAF	2.6	2.2	2.8	3.0	3.8	3.2	3.3	3.5		4.3
	$TC_{SAR>Lim}$	16.2	8.5	18.7	18.5	28.0	**30.4**	27.3	23.6		25.5
	THQ	0.9	0.9	1.0	1.0	**1.1**	1.1	1.0	1.1		1.0
	P_{TV}/V_{TV}	25.2	23.5	27.1	26.9	29.1	28.1	27.2	27.2		**29.3**
	# of excitations	2	2	2	2	1	2	2	1		1
**Pat Model 3**	$SAR_{max}(TV)$	59.8	76.4	77.2	82.3	92.6	**96.2**	93.2	83.7		90.4
	SAF	2.0	2.1	2.7	2.6	2.7	2.7	2.9	2.8		3.5
	$TC_{SAR>Lim}$	26.2	45.2	38.4	45.6	67.0	71.4	**76.0**	58.6		58.4
	THQ	0.9	1.1	1.2	1.2	**1.3**	1.3	**1.3**	1.3		1.2
	P_{TV}/V_{TV}	28.0	37.0	36.0	38.1	43.4	**44.8**	44.2	40.4		41.8
	# of excitations	2	2	1	1	1	1	1	1		1
**Pat Model 4**	$SAR_{max}(TV)$			50.0	49.4	49.0	51.5				**54.9**
	SAF			3.0	2.9	2.7	2.4				**3.2**
	$TC_{SAR>Lim}$			22.4	22.3	20.0	21.6				**34.1**
	THQ			0.9	0.9	0.9	0.9				**1.0**
	P_{TV}/V_{TV}			30.2	30.0	29.7	3.6				**33.0**
	# of excitations			1	1	2	2				1
**Pat Model 5**	$SAR_{max}(TV)$			44.0	46.5	**71.8**	48.5				52.5
	SAF			2.8	2.8	4.3	3.3				5.2
	$TC_{SAR>Lim}$			8.8	12.8	**42.2**	22.4				26.9
	THQ			0.9	1.0	**1.2**	1.0				1.0
	P_{TV}/V_{TV}			26.8	27.7	**38.2**	28.2				28.6
	# of excitations			2	2	1	1				1
**Pat Model 6**	$SAR_{max}(TV)$			**114.8**	83.1	100.1	80.9				
	SAF			4.6	**5.1**	5.8	4.9				
	$TC_{SAR>Lim}$			**65.8**	40.8	41.9	35.5				

	THQ	**1.5**	1.4	1.4	1.3
	P_{TV}/V_{TV}	**46.1**	37.9	40.2	36.1
	# of excitations	1	1	1	1
**Pat Model 7**	$SAR_{max}(TV)$	70.9	86.5	**91.2**	73.4
	SAF	4.0	4.4	4.3	**4.9**
	$TC_{SAR>Lim}$	44.3	61.0	**72.1**	41.9
	THQ	1.2	1.4	**1.4**	1.4
	P_{TV}/V_{TV}	34.0	39.7	**42.5**	33.8
	# of excitations	1	1	1	1
**Pat Model 8**	$SAR_{max}(TV)$	68.1	78.8	**86.3**	78.9
	SAF	**2.9**	2.7	2.4	2.5
	$TC_{SAR>Lim}$	28.5	**46.9**	37.2	36.8
	THQ	1.0	**1.1**	1.1	1.0
	P_{TV}/V_{TV}	29.6	**34.6**	32.4	32.6
	# of excitations	2	2	2	2
**Pat Model 9**	$SAR_{max}(TV)$	74.9	79.2	88.8	**93.9**
	SAF	2.2	2.3	2.5	**2.7**
	$TC_{SAR>Lim}$	44.8	49.1	60.2	**60.3**
	THQ	1.1	1.1	1.3	**1.3**
	P_{TV}/V_{TV}	37.8	39.5	44.3	**44.8**
	# of excitations	2	3	3	3

The highest value of each metric per patient model and optimization algorithm is highlighted in bold. The inter-algorithm maximum is further highlighted by green font. The overall (inter-algorithm and inter-patient) maximum is further highlighted by a green background.

Table S2. Full VOP uniformity optimization HTP results.

		VOP Uniformity Optimization									
		**8.P.R**	**16.P.R**	**16.I.R**	**16.I.E**	**16.2R.R**	**16.2R.E**	**32.2R.R**	**32.2R.E**	**16.I.R.WB**	**16.I.E.WB**
**Pat Model 1**	$SAR_{max}(TV)$	45.2	61.1	82.4	80.9	75.5	91.3	59.6	85.4	**101.1**	69.5
	SAF	1.8	2.1	2.4	2.6	2.6	3.0	2.5	**3.5**	3.0	3.0
	$TC_{SAR>Lim}$	3.2	20.5	47.5	45.8	**49.6**	46.7	31.9	43.7	29.7	29.9
	THQ	0.8	1.0	1.1	1.1	1.1	**1.2**	1.0	1.1	1.0	0.9
	P_{TV}/V_{TV}	24.5	29.5	36.0	36.2	36.0	**37.4**	32.0	36.1	31.7	33.8
	# of excitations	2	2	2	2	2	3	2	2	2	3
**Pat Model 2**	$SAR_{max}(TV)$	50.4	47.6	50.7	50.7	58.6	**59.5**	49.3	54.4		41.9
	SAF	2.3	2.5	3.1	2.9	3.8	3.1	2.8	3.4		**3.8**
	$TC_{SAR>Lim}$	13.3	8.9	13.9	12.9	**28.6**	25.9	13.7	20.5		0.3
	THQ	0.8	0.8	0.9	0.9	**1.0**	1.0	0.9	1.0		0.8
	P_{TV}/V_{TV}	22.6	21.1	24.8	25.3	**28.4**	26.2	23.1	25.4		23.9
	# of excitations	3	1	1	2	1	2	2	2		2
**Pat Model 3**	$SAR_{max}(TV)$	49.9	74.4	75.5	75.8	73.9	**82.7**	74.2	68.3		81.4
	SAF	2.2	2.0	2.3	2.4	2.4	2.3	2.3	2.5		**3.4**
	$TC_{SAR>Lim}$	11.1	43.3	44.1	43.3	44.2	**51.3**	48.7	36.7		46.8
	THQ	0.9	1.1	1.2	1.2	1.1	**1.2**	1.2	1.1		1.2
	P_{TV}/V_{TV}	22.4	36.8	37.1	36.7	37.0	**39.4**	37.6	34.6		38.7
	# of excitations	2	2	2	2	2	2	3	2		2
**Pat Model 4**	$SAR_{max}(TV)$			44.1	43.6	**47.2**	**47.2**				44.7
	SAF			2.5	2.3	2.3	2.1				**2.9**
	$TC_{SAR>Lim}$			5.4	2.7	**12.1**	10.4				6.6
	THQ			0.9	0.9	**0.9**	0.9				0.9
	P_{TV}/V_{TV}			28.1	27.2	**29.9**	29.0				28.3
	# of excitations			2	2	2	2				2
**Pat Model 5**	$SAR_{max}(TV)$			39.3	41.3	**54.1**	42.6				51.2
	SAF			2.3	2.2	2.9	2.7				**3.7**
	$TC_{SAR>Lim}$			0.0	0.7	**26.0**	2.7				10.6
	THQ			0.8	0.8	**1.0**	0.9				0.9
	P_{TV}/V_{TV}			23.4	24.0	**32.6**	26.9				28.2
	# of excitations			2	2	2	2				3
**Pat Model 6**	$SAR_{max}(TV)$			65.4	71.6	**88.7**	71.2				
	SAF			2.5	3.3	3.4	**3.4**				
	$TC_{SAR>Lim}$			30.9	36.3	**41.0**	37.7				
	THQ			1.0	**1.2**	1.1	1.1				
	P_{TV}/V_{TV}			32.6	36.6	**39.2**	36.5				
	# of excitations			2	2	2	2				
**Pat Model 7**	$SAR_{max}(TV)$			62.4	73.9	74.5	**74.6**				
	SAF			**3.8**	3.7	3.4	3.4				
	$TC_{SAR>Lim}$			37.9	46.7	56.1	**61.1**				
	THQ			1.2	1.2	**1.2**	**1.2**				
	P_{TV}/V_{TV}			32.0	35.0	36.6	**37.1**				
	# of excitations			2	2	2	2				
**Pat Model 8**	$SAR_{max}(TV)$			55.7	57.5	**67.1**	50.2				
	SAF			**2.1**	1.9	1.9	1.7				
	$TC_{SAR>Lim}$			**11.7**	6.4	10.7	7.8				
	THQ			0.8	**0.9**	**0.9**	0.8				
	P_{TV}/V_{TV}			24.4	23.7	**24.7**	**24.7**				

	# of excitations	3	3	2	3
**Pat Model 9**	SAR_{max}(TV)	53.3	53.0	**75.4**	71.9
	SAF	1.9	1.9	2.0	**2.1**
	$TC_{SAR>Lim}$	17.9	21.3	**53.5**	50.3
	THQ	0.9	0.9	**1.2**	1.1
	P_{TV}/V_{TV}	29.1	30.6	**41.0**	39.8
	# of excitations	3	3	2	3

Table S3. Full MVFS optimization HTP results.

		MVFS Optimization									
		**8.P.R**	**16.P.R**	**16.I.R**	**16.I.E**	**16.2R.R**	**16.2R.E**	**32.2R.R**	**32.2R.E**	**16.I.R.WB**	**16.I.E.WB**
**Pat Model 1**	SAR_{max}(TV)	59.5	74.0	104.6	114.6	119.5	**134.2**	112.9	121.9	112.4	80.0
	SAF	2.0	2.0	2.6	2.9	2.8	3.3	2.6	3.1	3.1	**3.7**
	$TC_{SAR>Lim}$	17.3	46.2	63.9	67.4	**83.5**	76.5	83.0	82.7	39.9	55.3
	THQ	0.8	1.1	1.2	1.3	1.5	**1.5**	1.4	**1.5**	1.1	1.1
	P_{TV}/V_{TV}	27.2	34.9	41.5	45.1	49.4	**66.8**	48.5	**51.5**	35.3	40.1
	# of excitations	2	2	2	2	2	2	2	3	1	2
**Pat Model 2**	SAR_{max}(TV)	53.1	51.4	55.6	62.0	74.0	75.5	81.8	**117.5**		55.0
	SAF	2.6	2.3	3.2	3.3	3.4	3.4	**3.9**	3.6		3.8
	$TC_{SAR>Lim}$	20.3	17.9	40.5	44.3	83.4	73.7	**84.0**	83.6		37.4
	THQ	0.9	0.9	1.0	1.1	1.3	1.2	1.3	**1.4**		1.0
	P_{TV}/V_{TV}	25.6	25.3	31.3	31.6	37.8	35.0	38.1	**40.0**		31.6
	# of excitations	2	2	2	2	2	2	2	2		2
**Pat Model 3**	SAR_{max}(TV)	66.1	81.5	98.2	90.9	104.3	100.8	**114.4**	109.1		99.3
	SAF	2.2	2.9	2.6	2.7	2.6	2.6	2.9	2.8		**3.4**
	$TC_{SAR>Lim}$	24.2	56.6	75.4	66.5	87.0	79.9	**96.9**	**94.2**		71.8
	THQ	1.0	1.1	1.3	1.2	1.4	1.3	**1.5**	1.4		1.3
	P_{TV}/V_{TV}	26.3	39.7	45.7	42.4	48.7	46.9	**53.1**	51.6		45.8
	# of excitations	3	2	1	2	2	2	2	2		1
**Pat Model 4**	SAR_{max}(TV)			58.2	58.3	59.9	**66.9**				61.3
	SAF			2.3	2.5	2.1	2.2				**3.1**
	$TC_{SAR>Lim}$			38.4	42.6	49.1	**50.8**				49.0
	THQ			1.0	1.0	1.1	**1.1**				1.1
	P_{TV}/V_{TV}			34.5	35.3	36.3	**37.4**				36.5
	# of excitations			3	2	3	2				2
**Pat Model 5**	SAR_{max}(TV)			52.4	56.2	**86.1**	66.2				58.5
	SAF			3.1	**3.7**	3.4	3.2				3.3
	$TC_{SAR>Lim}$			26.4	35.6	**76.5**	58.2				46.9
	THQ			1.0	1.0	**1.3**	1.1				1.0
	P_{TV}/V_{TV}			29.9	31.1	**47.7**	34.4				34.0
	# of excitations			3	2	3	2				3
**Pat Model 6**	SAR_{max}(TV)			126.4	112.3	**143.9**	119.0				
	SAF			**4.1**	3.7	3.7	3.1				
	$TC_{SAR>Lim}$			80.8	**83.1**	80.9	79.8				
	THQ			1.5	1.5	**1.6**	1.5				
	P_{TV}/V_{TV}			53.1	53.6	**56.8**	53.5				
	# of excitations			2	2	2	2				
**Pat Model 7**	SAR_{max}(TV)			87.8	107.0	97.8	**114.3**				
	SAF			3.8	3.9	3.5	**4.0**				
	$TC_{SAR>Lim}$			77.6	89.7	90.5	**95.8**				
	THQ			1.3	**1.5**	1.5	**1.6**				
	P_{TV}/V_{TV}			42.8	49.0	47.1	**52.8**				
	# of excitations			2	2	2	2				
**Pat Model 8**	SAR_{max}(TV)			95.2	107.5	**115.8**	90.5				
	SAF			2.6	**2.7**	2.5	2.5				
	$TC_{SAR>Lim}$			53.0	61.7	**63.1**	58.3				
	THQ			1.1	1.2	**1.2**	1.2				
	P_{TV}/V_{TV}			36.3	38.6	**41.0**	38.1				

	# of excitations	2	3	2	2
**Pat Model 9**	SAR_{max}(TV)	82.8	88.3	96.9	**99.7**
	SAF	2.3	2.3	2.4	**2.4**
	$TC_{SAR>Lim}$	56.7	64.8	**69.3**	69.0
	THQ	1.2	1.2	1.3	**1.3**
	P_{TV}/V_{TV}	41.5	44.5	48.3	**49.1**
	# of excitations	3	2	3	3

The highest value of each metric per patient model and optimization algorithm is highlighted in bold. The inter-algorithm maximum is further highlighted by green font. The overall (inter-algorithm and inter-patient) maximum is further highlighted by a green background.
